# Simultaneous heteroepitaxial growth of SrO (001) and SrO (111) during strontium-assisted deoxidation of the Si (001) surface†

**DOI:** 10.1039/d0ra06548j

**Published:** 2020-08-24

**Authors:** Zoran Jovanović, Nicolas Gauquelin, Gertjan Koster, Juan Rubio-Zuazo, Philippe Ghosez, Johan Verbeeck, Danilo Suvorov, Matjaž Spreitzer

**Affiliations:** Advanced Materials Department, Jožef Stefan Institute Jamova 39 1000 Ljubljana Slovenia zoran.jovanovic@ijs.si; Laboratory of Physics, Vinča Institute of Nuclear Sciences, National Institute of the Republic of Serbia, University of Belgrade Belgrade Serbia zjovanovic@vinca.rs; Electron Microscopy for Materials Science (EMAT), University of Antwerp Groenenborgerlaan 171 2020 Antwerp Belgium; Faculty of Science and Technology, MESA+ Institute for Nanotechnology, University of Twente P. O. Box 217 7500 AE Enschede The Netherlands; SpLine, Spanish CRG BM25 Beamline at the ESRF (The European Synchrotron) F-38000 Grenoble France; Instituto de Ciencia de Materiales de Madrid, Consejo Superior de Investigaciones Científicas (ICMM-CSIC) 28049 Madrid Spain; Theoretical Materials Physics, Q-Mat, CESAM, Université de Liège B-4000 Liège Belgium

## Abstract

Epitaxial integration of transition-metal oxides with silicon brings a variety of functional properties to the well-established platform of electronic components. In this process, deoxidation and passivation of the silicon surface are one of the most important steps, which in our study were controlled by an ultra-thin layer of SrO and monitored by using transmission electron microscopy (TEM), electron energy-loss spectroscopy (EELS), synchrotron X-ray diffraction (XRD) and reflection high energy electron diffraction (RHEED) methods. Results revealed that an insufficient amount of SrO leads to uneven deoxidation of the silicon surface *i.e.* formation of pits and islands, whereas the composition of the as-formed heterostructure gradually changes from strontium silicide at the interface with silicon, to strontium silicate and SrO in the topmost layer. Epitaxial ordering of SrO, occurring simultaneously with silicon deoxidation, was observed. RHEED analysis has identified that SrO is epitaxially aligned with the (001) Si substrate both with SrO (001) and SrO (111) out-of-plane directions. This observation was discussed from the point of view of SrO desorption, SrO-induced deoxidation of the Si (001) surface and other interfacial reactions as well as structural ordering of deposited SrO. Results of the study present an important milestone in understanding subsequent epitaxial integration of functional oxides with silicon using SrO.

## Abstract 2

A 1 nm-thick layer of SrO deoxidizes the silicon surface and experiences structural ordering into SrO (001) and SrO (111) epitaxial islands.

## Graphical abstract

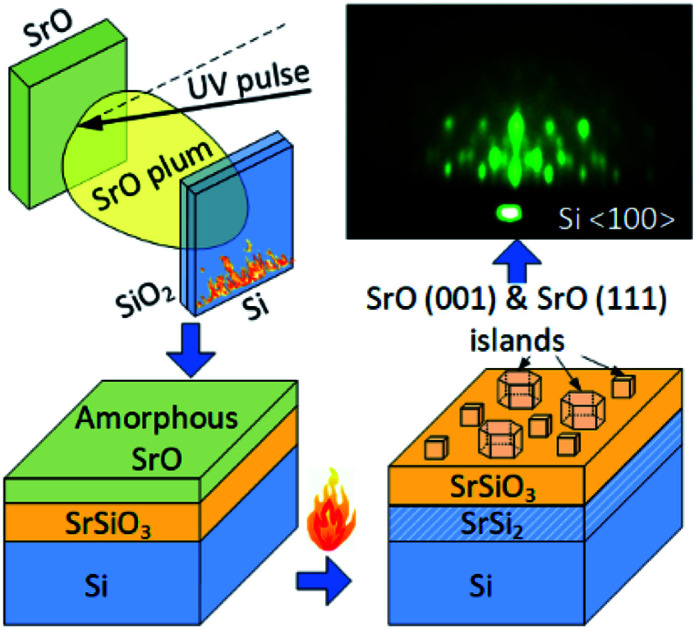

## 1. Introduction

Oxide materials are known for their diverse properties.^1,2^ Among them, complex oxides with a perovskite structure are promising candidates for epitaxial integration with a single crystalline silicon support.^3,4^ By unifying the intrinsic properties of complex oxides with the well-established processing for silicon, a novel platform with expanded functionality can be created.^5^ Although good matching of unit cells of perovskite-type complex oxides (SrTiO_3_ as an example) and silicon implies the viability of such integration, its realization is far from simple. The presence of a native oxide on the silicon surface as well as the high affinity of Si towards oxygen are the main reasons that hinder epitaxial integration of functional oxides with silicon.

Several traditional approaches based on wet chemistry^6^ and high temperature treatments^7^ are being used for deoxidation of the silicon surface, while state-of-the-art methods use Sr-/SrO assisted deoxidation under UHV conditions, achieving not only the removal of the native oxide but also an atomic-level control of the integration process. Previously, molecular beam epitaxy (MBE)^8–15^ and atomic layer deposition (ALD)^16,17^ methods were the only UHV methods capable of *in situ* deoxidation of Si and formation of a Sr-reconstructed silicon surface. In our recent studies, the same was achieved by the pulsed-laser deposition (PLD) method using strontium^18–20^ and strontium oxide.^21,22^

One of the key elements in epitaxial integration of functional oxides with silicon is the interface quality. However, due to the specific processing conditions, including a high temperature and an oxidizing environment, in combination with a substantial difference in the chemical properties of the materials, achieving control of the interface is challenging. An optimized MBE procedure has shown that 1–2 ML of Sr and 3–4 ML SrO are sufficient for simultaneous deoxidation and formation of a 2 × 1 Sr-reconstructed surface, which is suitable for epitaxial integration of SrTiO_3_.^10^ At other coverages, several surface reconstructions are possible, of which the most common are 3 × 2 (1/6–1/3 ML of Sr) and 2 × 1 (1/3–1/2 ML of Sr).^23–28^ In other cases, interfacing functional oxides with silicon is achieved by growing an appropriate buffer layer.^29–33^

The SrO has a long history of being used as a buffer layer for both Si (111) and Si (001). Machida *et al.* have shown that SrO grows on H-terminated Si (111) surface with epitaxial relationship SrO (111)‖Si (111) and SrO [1−10]‖Si [1−10], however, the quality of the epitaxial SrO layer decreased above 6 ML of SrO.^30^ In Niu *et al.* study the SrO has been grown at 150 °C at an O_2_ pressure of ∼4 × 10^−8^ torr on 2 × 1 Sr-reconstructed Si (001) surface. RHEED analysis has shown a cube-on-cube growth with clear indication of island growth.^34^ The cube-on-cube growth of SrO on silicon was also observed in Asaoka *et al.* study, while the SrO film became polycrystalline above 5 nm.^35^ In Tambo *et al.* study the evolution of 35 nm-thick SrO film on 2 × 1 reconstructed silicon surface was investigated as a function of annealing temperature in 10^−6^ torr O_2_ pressure. It was found that streaky RHEED patterns are stable up to 620 °C, while at 650 °C the pattern consisted only of the rings.^36^ The same was observed in the case of 10 nm-thick layer of SrO grown by MBE on 2 × 1 reconstructed silicon surface; streaky pattern, stable at 300 °C, was transformed at 500 °C into pattern characteristic for textured surfaces.^37^ Cube-on-cube growth of SrO on Si (001) surface appeared both in ref. 36 and 37. In Kado *et al.* study a 35 nm-thick layer of SrO was grown on chemically cleaned silicon surface. It was found that SrO grows with (100) SrO‖(100) Si and [011] SrO‖[001] Si orientation and that many extra sports were observed at the initial growth stages.^38^ In Higuchi *et al.* study TEM analysis has revealed a 6 nm-thick SrO film on top of amorphous Si-oxide layer, with orientation SrO (110)‖Si (100) and SrO [001]‖Si [011].^39^ Noteworthy, the stability of this pattern is thickness dependent: 50 nm thick SrO on Si was grown in a “cube-on-cube” manner *i.e.* SrO (100)‖Si (100) and SrO [011]‖Si [011].

The aforementioned examples illustrate the rich complexity of functional oxide–silicon interaction that includes not only the formation of silicate,^40–42^ silicide^43–45^ or carbide^18,46^ but also morphological changes such as formation of pits.^10^ To avoid this in the case of PLD method, the successful deoxidation of silicon surface with SrO requires careful control of experimental conditions and sub-unit cell thickness of SrO.^21^ If this is not the case, at slightly larger SrO thickness (1 nm) a spotty RHEED pattern, referred here to as ‘3D-structure’, appears.^22^ While having in mind the reaffirmed validity of the phrase: “the interface is the device”,^47,48^ it is important to understand the phenomena accompanying the integration of functional oxides with silicon.

From the cited literature about the epitaxial growth of SrO on Si (001) it can be concluded that the dominant epitaxial orientation was cube-on-cube growth. However, in certain cases a different epitaxial orientation was observed. Higuchi *et al.* have shown that epitaxial orientation of SrO to silicon can be described as SrO (110)‖Si (100) and SrO 〈001〉‖Si 〈011〉.^39^ Also, it was found that this epitaxial orientation was thickness dependent. Besides, the same study reported additional transmission spots the origin of which was not discussed by authors. In our previous studies,^21,22^ the 3D structure was reported for the first time for SrO–silicon system, however without detailed analysis. In the present study we aim to provide novel insight into structural ordering at the surface/interface that is driven by deoxidation process. We carefully analyzed the chemical and structural properties of 3D structure by combining scanning transmission electron microscopy (STEM), electron energy loss spectroscopy (EELS), synchrotron X-ray diffraction (XRD) and reflection high energy electron diffraction (RHEED) methods. The results are showing that the 3D-structure, appearing during silicon surface deoxidation induced by pulsed laser deposited SrO, is a consequence of a complex interplay between SrO desorption, SrO-induced deoxidation of Si (001) surface and epitaxial structural ordering of SrO deposited on silicon surface.

## 2. Experimental details

### 2.1. Sample pre-treatment

The substrate (5 × 5 mm^2^ B-doped Si(100), Si-Mat, Germany) was ultrasonically cleaned in acetone and EtOH for 3 min, respectively, thoroughly rinsed with EtOH and blow-dried with a N_2_ gun. Subsequently, the substrate was glued to a stainless-steel sample plate using silver paste (Leitsilber 200, Ted Pella, Inc., USA). Prior to insertion into the PLD chamber (Twente Solid State Technology, Netherlands) the sample plate, with the Si substrate, was heated up in air (∼120 °C) to remove the organic solvent present in the paste. Once inserted into the PLD chamber, the sample was degassed at 650 °C for 1.5 h in vacuum (2 × 10^−8^ mbar) followed by a 30 min treatment in 1–1.5 × 10^−5^ mbar O_2_ at 600 °C to minimize carbon contamination.

Heating was achieved using an IR laser (*λ* = 800–820 nm, HighLight FAP 100, Coherent, USA) coupled with an IMPAC IGA 5 pyrometer (LumaSense Technologies, Inc., USA) with an 85% emissivity constant. In our previous work,^22^ the sample was heated using resistive heater and a thermocouple was measuring the core of the resistive heater, while in present study the sample was heated by laser heating and the temperatures were acquired from the sample surface using a pyrometer. The surface of the sample was monitored *in situ* using RHEED (KSA400, STAIB instruments, Germany), while KrF excimer laser (*λ* = 248 nm, 25 ns, COMPexPro 205 F, Coherent, Germany) was used for the (pre)ablation of the SrO and TiO_2_ single-crystalline target (SurfaceNet, Germany).

### 2.2. SrO-assisted deoxidation of the silicon surface

**Fig. 1:**
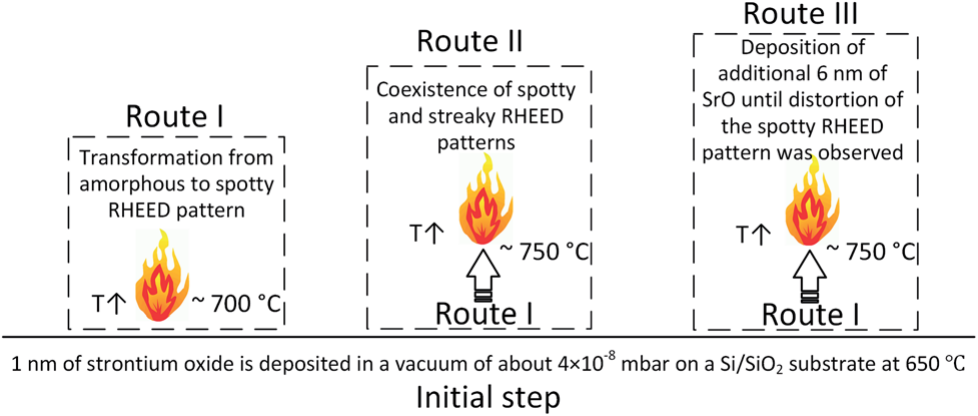
Schematic representation of routes used for synthesis of samples.

In our previous works,^21,22^ the effect of SrO thickness on deoxidation of silicon surface was studied. It was established that deoxidation process requires careful control of SrO thickness to produce smooth, oxide-free and Sr-passivated silicon surface. During this process, a spotty RHEED pattern *i.e.* 3D structure as characteristic feature appeared when ∼1 nm of SrO was used for native oxide removal. The present study is focused on analysis of 3D structure, while previous works examined wider framework of SrO–Si reaction in the case of PLD method. For analysis of 3D structure, we have developed procedures based on the specific processes occurring in the samples. Namely, due to sensitivity of SrO to environmental conditions (moisture and CO_2_) a protective TiO_2_ capping layer was applied. This was the case for all *ex situ* analyses (Fig. 1). Route I represent the case when 3D structure is well defined. The sample prepared *via* route I was analyzed *in situ* by RHEED and *ex situ* by synchrotron XRD. The aim was to study the 3D structure in it's the most characteristic state. Based on this results, and route II and route III were designed. Route II was dedicated to *in situ* RHEED analysis of 3D structure while 2 × 1 Sr-reconstruction is simultaneously present. It is important to emphasize that the amount of deposited SrO in route I and II was ∼1 nm. Next, in route III, additional 6 nm of SrO was deposited on 3D structure to understand its epitaxial orientation to silicon and interfacial processes.

**Fig. 2:**
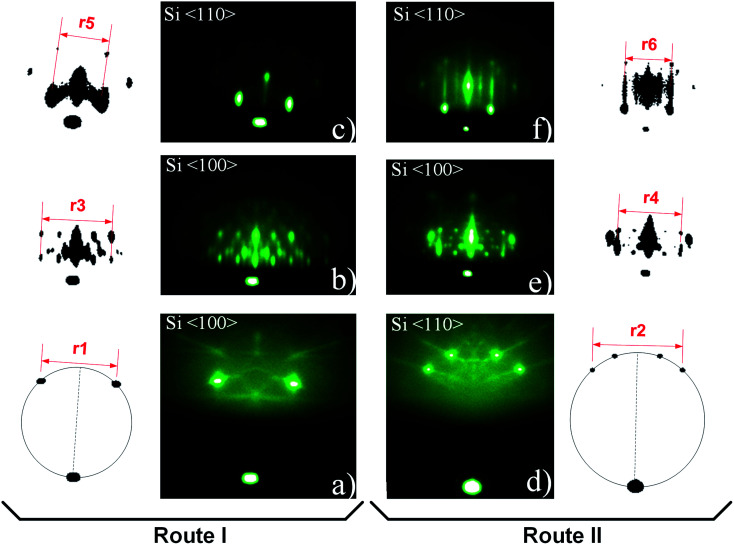
RHEED patterns of Si (001)/SiO_2_ substrate (a and d), 3D pattern (b and c) and mixture of a 3D pattern and 2× (1×) Sr-reconstruction of silicon surface (e and f). For clarity, RHEED features are shown separately and the characteristic segment length is marked (*r*1–*r*6). The azimuthal direction is marked in the upper left corner of RHEED images.

The common part of all routes included deposition of 1 nm of strontium oxide in a vacuum of about 4 × 10^−8^ mbar on a Si/SiO_2_ substrate at 650 °C.^21,22^ The fluency, repetition rate, spot size and target-to-substrate distance were 1.3 J cm^−2^, 0.1 Hz, 2.31 mm^2^ and 5.5 cm, respectively. In route I, the temperature was increased to 700 °C by 20 °C min^−1^ when a well-defined spotty RHEED pattern, characteristic of 3D-structure, appeared (Fig. 2b and c). In the route II the temperature was increased to 750 °C with 20 °C min^−1^ and kept for 2–3 min until a 2 × 1 Sr-reconstruction of Si (001) surface appeared (co-exists with 3D-RHEED pattern, Fig. 2e and f). In route III, a sample with a larger number of SrO pulses was prepared. Initially, 1 nm of SrO was deposited on a Si/SiO_2_ substrate at 650 °C and vacuum of about 4 × 10^−8^ mbar and heated at a rate of 20 °C min^−1^ until a well-defined characteristic spotty RHEED pattern was formed (∼700 °C). Next, temperature was increased to 750 °C with 20 °C min^−1^ and deposition of SrO was continued in 1.2 × 10^−2^ mbar Ar with 0.5 Hz rate, until a first indication of distortion of the spotty RHEED pattern was observed (∼7 nm total thickness of SrO, Fig. S1, ESI†). The higher deposition rate of SrO in route III was used to minimize deterioration of RHEED pattern due to concurring SrO desorption and SiO_2_/Si deoxidation that might influence the stability of 3D structure at higher temperatures.

Because of SrO reactivity to environmental conditions, for *ex situ* analyses the samples were protected with TiO_2_ capping layer. The sample was cooled to ∼100 °C, after which 300 pulses of TiO_2_ were deposited with 1 Hz in 0.13 mbar O_2_, followed by 300 pulses in 0.05 mbar and 1000 pulses in 0.012 mbar O_2_. The fluency, spot size and target-to-substrate distance were kept the same as described above. The sample prepared by the route I was examined using XRD at The Spanish CRG Beamline BM25-SpLine^49^ at the ESRF – The European Synchrotron – using a six-circle diffractometer in vertical geometry, a photon energy of 15 keV and a beam spot size of 0.3 × 0.3 mm^2^. A high-resolution multi-pixel 2D detector was used to acquire the spectra in a short time to reduce the radiation damage on the ultra-thin layer. The XRD diffractograms were obtained at RT and under a flow of nitrogen to prevent from deposition of ozone on the sample surface due to the interaction between air and the intense beam of X-rays.

The TiO_2_-capped sample obtained in route III was transported to the EMAT laboratory in Antwerp, Belgium for TEM analysis. Within the glove box, the sample was placed into a Kammrath & Weiss GmbH transfer module which possesses a one way valve that does not allow the inflow of gas from the outside, together with the Omniprobe copper support grid and then transferred to the FIB (FEI Helios 650).^50–55^ The FIB sample was prepared as described elsewhere^56^ and transferred without exposure to air into a Gatan double-tilt vacuum transfer holder for TEM investigation using a FEI Titan 60–300 microscope with an X-FEG high brightness electron source, a probe Cs corrector, a Super-X 4-quadrant EDX detector and a Gatan GIF Enfinium electron energy loss (EEL) spectrometer. The microscope was operated in scanning TEM mode at 120 kV and 10 pA to minimize damage to the film. Imaging was performed with a 21 mrad convergence angle and collection of all electrons in the range 46–160 mrad for high angle annular dark field (HAADF). Core-loss and low-loss EELS measurements were performed with 20 pA beam current.

## 3. Results and discussion

### 3.1. STEM and XRD analysis

**Fig. 3:**
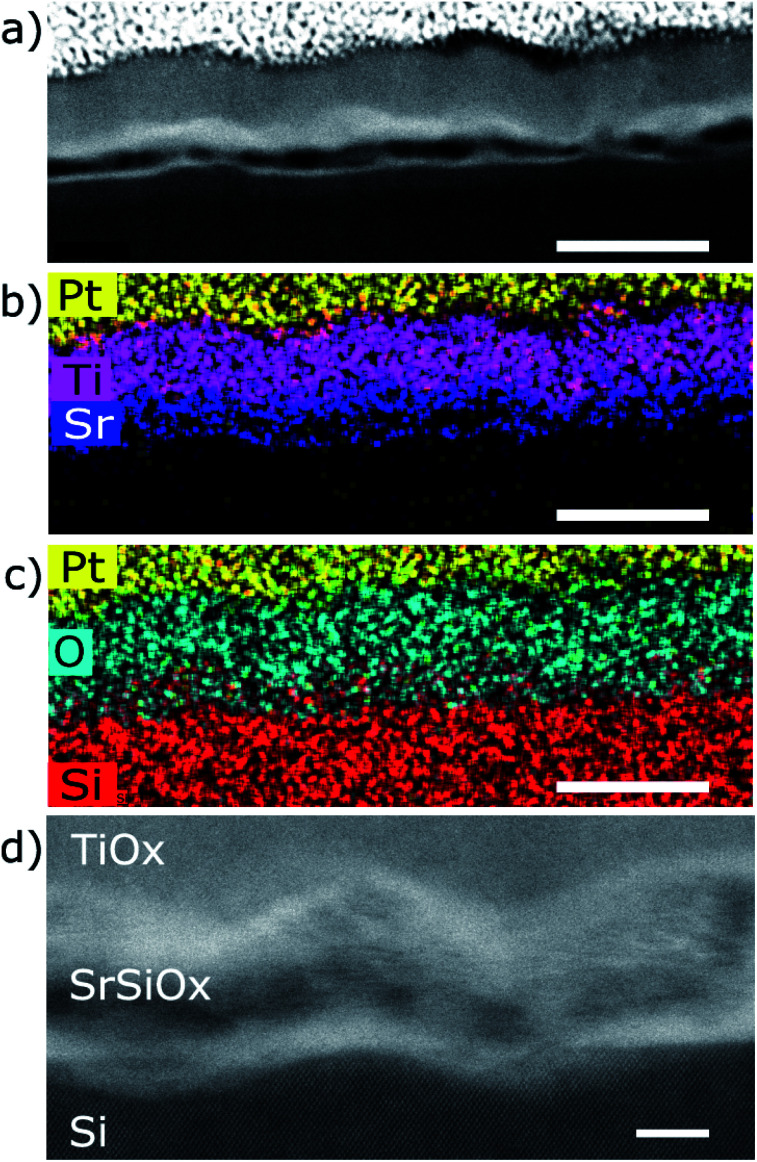
STEM HAADF images of Si/SrSi_*x*_/SrSiO_*x*_/SrO_*x*_/TiO_*x*_ sample. (a) Low magnification HAADF-STEM image of the region where EDS was performed showing the roughness of the film. (b) EDS map of Pt, Ti and Sr showing a good definition of the layers. (c) EDS map of Pt, O and Si showing the presence of O and Si together in the SrSiO_*x*_ layer. Scale bar in (a–c) is 40 nm. (d) Higher resolution image showing crystallinity of the Si substrate, while it is absent in other layers. Scale bar in (d) is 5 nm.

The STEM analysis revealed that the sample prepared in route III consists of a 28 nm bilayer film (16 nm TiO_*x*_ on top of 12 nm SrO_*x*_) grown on Si substrate (Fig. 3). The HAADF-STEM image is showing a wavy interface between the layers (Fig. 3d) which is consistent with the observed 3D RHEED pattern. Based on TEM analysis the roughness can be estimated to ∼3 nm at the SrO_*x*_–Si interface and 7–10 nm on the TiO_*x*_–SrO_*x*_ interface.

**Fig. 4:**
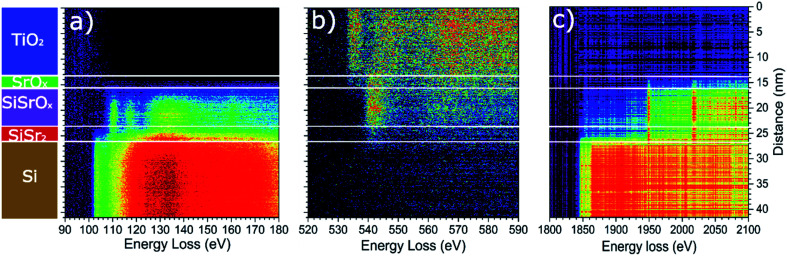
EELS line profile of the (a) Si L_2,3_ edge, (b) O K edge and (c) Si K and the Sr L_2,3_ edges. On the left a schematic of the layer stacking is displayed for easier understanding. We can notice the presence of the thin SrSi_*x*_ layer (no oxygen signal) due to deoxidation of the Si and the presence of a thin layer of SrO_*x*_ at the interface between SrSiO_*x*_ and TiO_2_.

EELS line-scans and the HAADF intensity profile of the sample acquired simultaneously are shown in Fig. S2 of the ESI.† The analysis of the EELS line profiles of the Si K and Sr L edge revealed that most of the SrO_*x*_ layer is a strontium silicate with only a 2–3 nm thick layer of SrO at the interface with TiO_*x*_ (Fig. 4). This is confirmed by the EELS line profiles of Si L and O K-edge (Fig. 4a and b).

A more detailed examination of the layered structure has shown that SrSiO_*x*_ layer is very sensitive to electron-beam damage. This hindered the examination of the crystallinity of the SrSiO_*x*_ layer (Fig. S3a, ESI†). Furthermore, after exposure of the TiO_*x*_/SrO_*x*_/SrSiO_*x*_ interfaces to the electron beam, formation of a crystalline perovskite phase of SrTiO_3−*x*_ was observed (Fig. S3b, ESI†). Nonetheless, a thin and oxygen-free (since no oxygen is detected in this region in the EELS profile on Fig. 4b), Sr-containing layer at the Si interface appeared to be less sensitive to the electron beam, which might indicate a crystalline silicide in the first atomic layers near the silicon substrate.

**Fig. 5:**
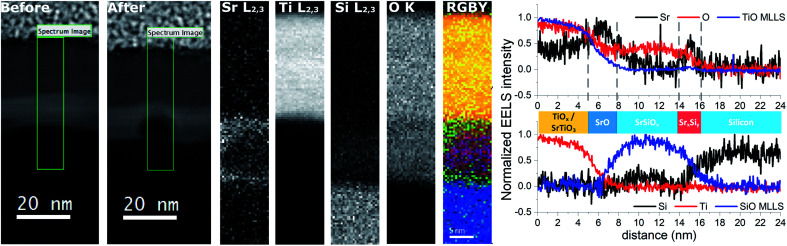
Elemental EELS mapping of the Si/SrSi_*x*_/SrSiO_*x*_/SrO_*x*_/TiO_2_ sample. From left to right are represented: the areas before and after the measurement showing the sensitivity of the SrSiO_*x*_ layer to the electron beam; the extracted 2D elemental maps of the Sr L_2,3_, Ti L_2,3_, Si L_2,3_ and O K edges. Subsequently, in the RGBY are superimposed with color codes O in red, Sr in green, Si in blue and Ti in yellow. On the right the top panel shows the TiO_*x*_/SrTiO_3_ fine structure MLLS fitting of the O K edge (identical profile to the Ti L_2,3_ edge, shown in the bottom panel) together with the Sr L_2,3_ edge and the full O K edge, showing clearly the presence of an SrO layer at the interface of the TiO_*x*_/SrSiO_*x*_ interface. Bottom panel shows the Ti L_2,3_ edge, the full Si K edge and the SrSiO_*x*_ fine structure MLLS fitting of the O K edge showing clearly the presence of a strontium containing layer at the interface of Si and SrSiO_*x*_ resulting from SrO-assisted deoxidation of Si. This shows clearly the presence of a 2 nm SrO layer at the interface between SiO and TiO as well as migration of Sr at the interface between Si and SiO.

Two dimensional maps of the O K edge, Si K edge, Sr L_2,3_ and Ti L_2,3_ edges show the elemental distribution of O, Si, Sr and Ti in different layers, and like in previous cases, a high-quality mapping of the selected area was not possible due to the sensitivity of the SrSiO_*x*_ to the e-beam (Fig. 5). Nonetheless, the results are showing clear differentiation between the layers with different distribution of strontium as well as the absence of oxidation of the silicon substrate. As can be seen, a higher concentration of strontium is located near the Si substrate, which can be ascribed to strontium-assisted deoxidation process. At the same time, silicon was observed away from the Sr–Si interface suggesting Si diffusion from the substrate during the deoxidation process. We see on both profiles a clear layer of 1.5 nm containing Sr at the interface between Si and SiO. In the SiSrO_*x*_ layer, Sr is just present as dopant, the amount of interdiffusion cannot be determined due to the roughness of the interface.

The presence of a stronger contrast in the Sr L_2,3_ map in the SrSi_*x*_ and SrO layers around SrSiO_*x*_ and the formation of a hole inside the SrSiO_*x*_ layer are linked to the high reactivity of SrSiO_*x*_ and its amorphous character. In fact, we can infer from the HAADF contrast in the after image that some Sr is migrating away from the substrate because of beam induced diffusion. TEM analysis infers that the roughness at the TiO_2_ interface (7–10 nm) is higher than that of the Si interface (∼3 nm, Fig. 3). As explained by Wei *et al.* the optimization of the Sr/SrO amount for silicon native oxide removal is of paramount importance for preparation of high quality interface; if this is not the case, surface pits occur.^10^ Thus, an increased roughness at the Si interface can be a consequence of uneven deoxidation reaction. On the other hand, a higher roughness at the TiO_2_–SrO interface is indicative of island growth mode *i.e.* surface migration and agglomeration of the deposited SrO.

Another important aspect is the thickness of the silicate layer (∼10 nm, Fig. 3). It has been shown previously that deposition of 1 nm of SrO at 650 °C (0.1 Hz deposition rate) on Si/SiO_2_ is accompanied by 1.5 nm of strontium silicate.^22^ In the case of route III (Fig. 3–5), deposition of additional 6 nm of SrO was performed at 750 °C, with 0.5 Hz deposition rate. Based on the SrO deposition time a proportional increase of SrO thickness should be expected. However, prolonged deposition of 6 nm-thick layer of SrO on Si at high temperature promotes two processes: desorption of SrO and SrO–Si reactions. Consequently, thinner layer of strontium oxide (2.5 nm) and thicker layer of silicate (due to SrO desorption and an enhanced formation of the silicate^22^) was observed.

The EELS analysis revealed the presence of a SrO layer on top of the silicate layer, thus confirming previous findings of angle-resolved XPS.^22^ However, as the interfacial layer was very sensitive to e-beam it was not possible to perform detailed structural analysis. Therefore, we prepared a sample using the single step method (route I), also protected with a TiO_2_ capping layer, and characterized it by using synchrotron XRD.

**Fig. 6:**
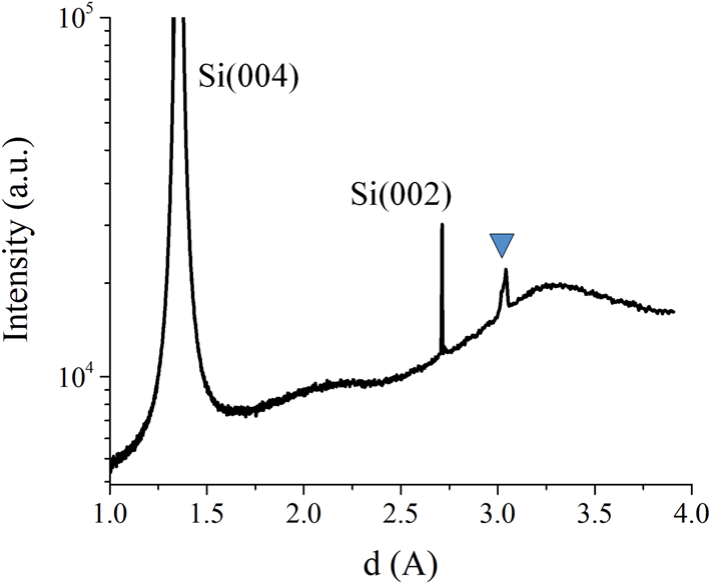
XRD pattern of Si/SrSi_*x*_/SrSiO_*x*_/SrO_*x*_/TiO_2_ sample. The triangle marks the strongest peak belonging to the deposited material *via* route III.

The Fig. 6 shows a diffraction peak at *d* ∼ 3.03 Å, which is close to the theoretical *d*-spacing of SrO (111) plane (*d* = 2.979 Å). Based on possible reactions between Sr, Si and O, there are several candidates that could contribute to XRD peak at *d* ∼ 3.03 Å (Table 1). The possible candidates were not considered based on XRD information only. Transmission RHEED patterns, as shown in Tables 2 and 3, clearly identified √2 relationship between characteristic features (also along 〈100〉 and 〈110〉 azimuths), which is characteristic of cubic systems so that metallic Sr or Si_2_Sr could be possible candidates. However, since both XPS^22^ and EELS (Fig. 4 and 5) suggested SrO in the topmost layer, we considered SrO as more probable candidate that contributes to spotty RHEED pattern.

**Table 1:** The crystallographic parameters of possible Sr-, Si- and O-containing candidates

	Sr	Sr_2_Si	Sr_5_Si_3_	SrSi	Sr_3_SiO_5_	Si_2_Sr
CoD^a^	9008484	1520913	8101453	1538529	4001112	1536083
SG^b^	*Fm*3̄*m*	*Pnma*	*I*4/*mcm*	*Cmcm*	*P*4/*ncc* [origin 2]	*P*4_3_32
*a* (Å)	6.085	5.162	8.089	4.830	6.951	6.535
*b* (Å)		8.133		11.330		
*c* (Å)		9.544	15.733	4.040	10.761	
Plane	(002)	(211)	(123)	(111)	(121)	(012)
*d*-Spacing (Å)	3.042	3.028	2.978	2.989	2.987	2.923

a Crystallography Open database ID.

b Spacegroup.

**Table 2:** Reciprocal space distances of characteristic segments in RHEED patterns of 3D-structure and 2 × 1 Sr-reconstructed surface

	Route I		Route II			
Figure	2b	2c	2e	2f	2e	2f
App^a^	Spotty		Spotty		Streaky	
Az^b^	〈100〉	〈110〉	〈100〉	〈110〉	〈100〉	〈110〉
SL^c^	*r*3/4	*r*5/2	*r*4/4	*r*6/2	*r*4/2	*r*6/4
rSL^d^	0.351	0.497	0.358	0.512	0.716	0.256

a Appearance.

b Azimuth.

c Segment length.

d Segment length in reciprocal space, in Å^−1^.

**Table 3:** Distances of the dashed-line rectangle in Fig. 7

Label	*r*3/4	*l*1 (√2(*r*3/4))	*l*2	A–B	C–D
Distance/Å^−1^	0.351	0.497	0.127	0.096	0.126

The asymmetry of the XRD peak at *d* ∼ 3.03 Å suggests the presence of two sets of planes with close interplanar spacing. Stoichiometry of the SrO can be easily changed due to concurrent process of deoxidation in which, in the first step, the oxygen is taken from SiO_2_ and later from SrO, thus leaving Sr-reconstructed surface. We suppose that deoxidation process might not proceed in an even manner across the surface thus leaving SrO with different amount of oxygen that would lead to different in-plane distance. To obtain additional insight, a more detailed structural analysis was based on *in situ* RHEED method.

### 3.2. Analysis of RHEED patterns

To examine RHEED patterns of 3D-structure (route I) and 2 × 1 Sr-reconstructed silicon surface (coexisting with 3D pattern, route II) we used the high-symmetry azimuthal directions and screen constants relative to the known crystallographic properties of Si substrate (Fig. S4, ESI†). The screen constants for route I and II (0.0789 Å^−1^ and 0.0781 Å^−1^, respectively) were calculated from distances *r*1 and *r*2 in Fig. 2a and d. The RHEED patterns of 3D structure and 2 × 1 Sr-reconstructed surface were acquired at the characteristic temperatures (700 and 750 °C) and corresponding unit cell sizes were calculated from these patterns. The distances were measured between the clearly defined spots *i.e.* from their center defined by the highest intensity of light.

In Fig. 2 RHEED patterns appear as spotty (route I, Fig. 2b and c) and a combination of spotty and streaky (route II, Fig. 2e and f), thus indicating the presence of three-dimensional protrusions on a smooth two-dimensional surface. For clarity, the most important features of RHEED patterns are shown next to corresponding images and were used for calculation of reciprocal space distances. See ESI† for calculation details.

Let us first consider the RHEED pattern obtained in route I (Fig. 2b). The length of the characteristic segment in the reciprocal space can be obtained when the screen distance (*r*3) is divided by the number of segments (*n* = 4), and multiplied by screen constant, which is equal to 0.351 Å^−1^. Following the same approach, the length of the segment can be calculated also for other RHEED patterns (Table 2). It can be observed that the distance between the streaks of 2 × 1 Sr-reconstructed silicon surface (Fig. 2e and f) matches nicely the theoretical distances of 2 × 1 reconstructed Si (001) (Fig. S5a, ESI†).

**Fig. 7:**
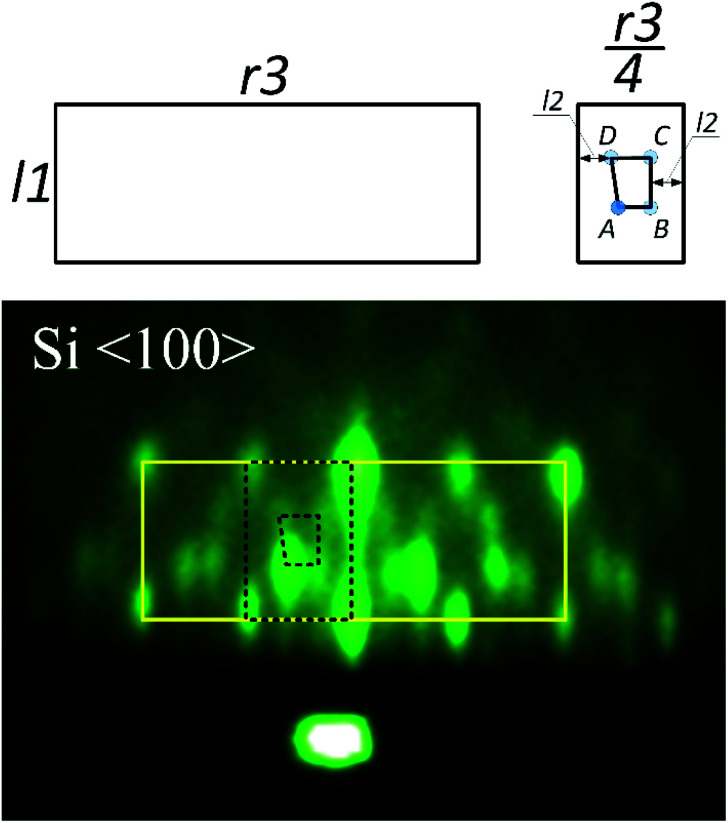
RHEED pattern of 3D structure. The solid line marks four the most intense rectangular features, one of which is represented by dashed line. Note that the RHEED pattern reveals mirror like symmetry. For clarity, the characteristic distances are schematically shown on top. The pattern was acquired in route I.

Fig. 7 shows a spotty RHEED pattern in which a 4-segment structure can be identified. This structure is clipped out and schematically shown together with characteristic distances (*l*1, *r*3). Our analysis shows that *l*1 is approximately equal to √2(*r*3/4). Furthermore, within this structure we can identify a segment, marked by dashed line, with four inner spots (A–D), whose distances are given in Table 3.

**Fig. 8:**
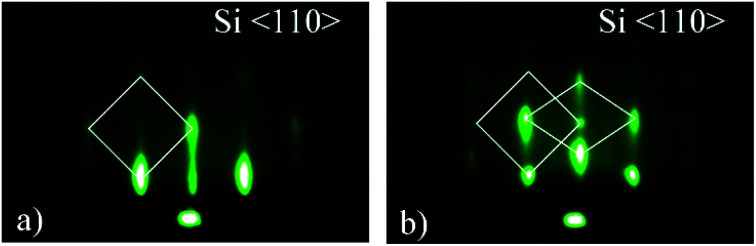
RHEED patterns of the 3D-structure viewed along Si^〈110〉^ azimuth showing (a) square-shape transmission pattern and (b) square- and rhomb-shape pattern at a bit higher incident angle. The patterns were acquired in route I.

The pattern obtained along the Si^〈110〉^ azimuth is shown in Fig. 8. Fig. 8a shows a square-shape pattern, beside which a rhombohedral structure can be observed at slightly higher tilt angle (Fig. 8b). The pattern in Fig. 8 can be interpreted as the superposition of two crystalline orientations. Because of multiple candidates (silicides and silicates), the identification based on RHEED pattern was impossible without the additional results of other methods (EELS, XRD, XPS). The possible candidates for 3D structure are Sr-silicate and/or Sr-silicide, however since XPS results^22^ and the EELS (Fig. 4 and 5) strongly suggest SrO as the topmost layer, whose crystallinity was additionally corroborated by XRD results (Fig. 6), different SrO crystallographic orientations should be considered as the most probable ones. The most likely contribution of SrO to 3D pattern is additionally corroborated by good agreement (∼8% difference) of the measured reciprocal space distances (Table 2) with the reciprocal space distances of SrO viewed along [100] zone-axis (Fig. S5b, ESI†). This relatively large discrepancy will be discussed later.

**Fig. 9:**
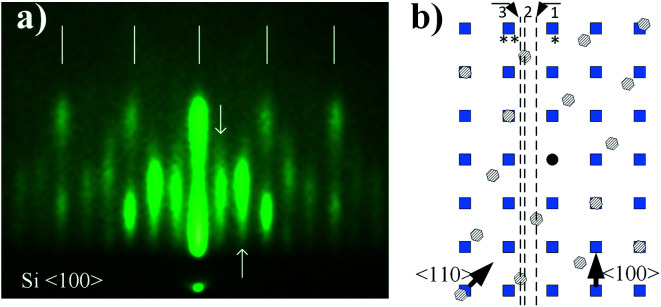
(a) The RHEED pattern in the initial stages of 3D structure formation. (b) Superimposed lattices of SrO (square – (100) surface, hexagon – (111) surface). The reciprocal lattice of SrO (111) is rotated for 15 degrees in respect to SrO (100). Arrows in (b) indicate azimuth directions of Si (001) substrate. The distance between lattice points is scaled accordingly. The pattern is characteristic for all routes, since it appears in the initial stages of 3D structure formation.

Since the results of other experimental methods suggest SrO as the topmost layer, to understand contribution of different crystallographic orientations to 3D pattern, it is important to examine it in its initial stages (Fig. 9a). As can be observed, the streaky character of the pattern suggests a more two-dimensional character in the initial stages of its formation.

Let us consider reciprocal lattices of SrO (100) and (111) (Fig. 9b), where the latter one was rotated in-plane by 15 degrees (to be explained later). Considering the RHEED pattern in Fig. 2, in the Fig. 9b we can identify Si^〈100〉^ and Si^〈110〉^ azimuths, a distance between the lines 1–2 and 1–3 and their position in respect to diffraction points marked as * and **. Distance of line 1 to * and 2 to ** is approximately the same as *l*2, while the distances between lines 1–2 and 1–3 are 0.094 Å^−1^ and 0.128 Å^−1^, respectively, which is in good agreement with A–B and C–D distances (Table 3).

At the same time, there is a good agreement when RHEED pattern (Fig. 8) and superimposed reciprocal lattice (Fig. 9b) are viewed along Si^〈110〉^ azimuth, since all diffraction points are collinear and equally spaced (0.548 Å^−1^, which agrees well with the experimental value ∼ 0.50 Å^−1^, Fig. 2). This clearly reveals that reflection from smooth flat surfaces of SrO (100) and (111), present in the initial stage, contributes to observed RHEED pattern (Fig. 9a).

**Fig. 10:**
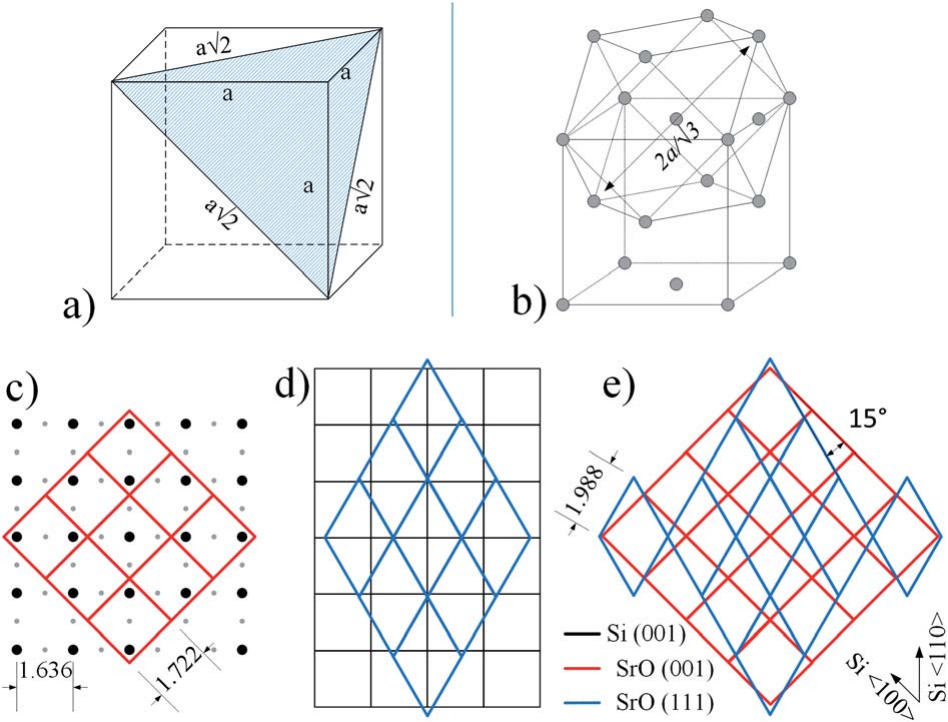
(a) The unit cell of SrO crystal with marked (111) plane; (b) crystal structure of SrO presented in form of (100) and (111) growth orientation. (c) SrO (100) growth on 2 × 1 reconstructed Si (001) surface; (d) SrO (111) growth on Si (001) (for clarity 2 × 1 reconstruction is not shown); (e) relationship of SrO (100) and SrO (111) grown on Si (001). The unit cell sizes are scaled accordingly.

We will now turn to the origin of the 15° offset between reciprocal lattices of SrO (111) and SrO (100), by focusing on the real space growth of SrO on Si (001). Let us consider the unit cell of SrO crystal with highlighted (111) plane (Fig. 10a). The (111) plane with respect to the cube's edges forms a triangular pyramid of height *a*/√2 and base length *a*√2. As it is known, the unit cell sizes of Si and SrO are 5.43 Å and 5.16 Å, respectively, which means 4.97% mismatch in the case of cube-on-cube growth. However, growth of SrO (100) on Si (001) is possible to occur also when SrO lattice is rotated for 45° to match the substrate (Fig. 10c), which significantly reduces mismatch. However, in the case of (111) out-of-plane orientation of SrO, the length of the base edge is *a*√2, for which epitaxy is preferable if four unit cells of Si (001) are matched by three unit cells of SrO (111) (Fig. 10d). The overlap of the two SrO structures (Fig. 10e) clearly explains the 15° offset that was observed. This indicates that SrO grows both in (001) and (111) out-of-plane orientation on Si (001). Also, elongation of the spots of the RHEED patterns (Fig. 7 and 8) is showing that SrO islands have high aspect ratio *i.e.* the width is much larger than the height of the islands.^57^

In Chen *et al.* and Higuchi *et al.* studies the SrO has been used both as buffer layer and deoxidizing agent.^39,58,59^ It was observed that during heating of thin SrO layer on Si/SiO_2_ a characteristic RHEED pattern is formed, however, only at certain temperature (∼700 °C). TEM analysis has revealed a 6 nm-thick SrO film on top of amorphous Si-oxide layer, with orientation SrO (110)‖Si (100) and SrO 〈001〉‖Si 〈011〉.^39^ Noteworthy, the stability of this pattern is thickness dependent: 50 nm thick SrO on Si was grown in “cube-on-cube” manner *i.e.* SrO (100)‖Si (100) and SrO 〈011〉‖Si 〈011〉. It was also highlighted that SrO (110)‖Si (100) and SrO 〈001〉‖Si 〈011〉 orientation appeared only when native oxide was present, as in our case. This might imply that silicate character of the layer in intimate contact with SrO influences its epitaxial orientation.

In the case of our samples, the layer structure observed by TEM is the same for all three routes, with main difference in their thickness. In route III an additional 6 nm of SrO was deposited on well-defined 3D structure. Because of deposition at higher temperature, desorption of SrO occurred as well as reaction with silicon to silicates. This resulted in thinner SrO and thicker silicate layer than expected. The route II should contain less silicate than route I since part of it was removed and 2 × 1 Sr-reconstruction appeared. So, in the terms of SrO and silicate thickness it is: route III > route I > route II. In route II, the silicate is located beneath 3D structure, while in deoxidized region the appearance of 2 × 1 Sr-reconstruction is clear indication of its absence. Finally, this influences the orientation of SrO on Si (001). The common cube-on-cube growth of SrO on Si (001) would be present in the case of atomically sharp interface between the two materials. However, in our case the RHEED analysis suggests different epitaxial relationship, which we attributed to the presence of interfacial silicate.

Based on distances in the reciprocal space the unit cell size of SrO can be calculated. The values obtained from Fig. 2e and f are more illustrative due to simultaneous presence of 2 × 1 Sr-reconstruction of silicon surface and spotty pattern. The unit cells size of SrO (5.58 ± 0.06 Å and 5.53 ± 0.06 Å for Si^〈100〉^ and Si^〈110〉^ azimuths, respectively) and overlapping of the main RHEED features of 3D-structure and 2 × 1 Sr-reconstructed Si surface, indicate SrO adaptation to silicon substrate.

When comparing the reciprocal space distances of 2 × 1 Sr-reconstructed silicon surface (Fig. 2e and f), with theoretical ones, a difference of 2.98 and 1.92% can be observed for Si^〈100〉^ and Si^〈110〉^ azimuth, respectively. In the case of 3D pattern, occurring simultaneously with the 2 × 1 Sr-reconstruction (Fig. 2e and f), the reciprocal space distances differ from the theoretical values of SrO for 7.73 and 6.57% along Si^〈100〉^ and Si^〈110〉^ azimuth, respectively. Noteworthy, determination of screen constant shows that the intrinsic experimental error (∼1%) should be accounted for in the differences. The fact that experimental values of 2 × 1 Sr-reconstructed silicon surface are ∼2–3% larger than the theoretical ones, indicates probable presence of other adatoms, such as oxygen, that might influence the size of surface unit cell. In case of SrO, such large difference would be expected for cube-on-cube growth of SrO (001) on Si (001) (∼4.97% mismatch); however, mismatch in cases shown in Fig. 10c and d is significantly lower (<1%). The possible reason for larger unit cell, observed by XRD and RHEED methods, might be not only the atomic arrangement of the Sr-reconstructed surface, but also different thermal expansion coefficients of SrO and Si. Namely, for SrTiO_3_ films on Si substrates it has been demonstrated that in relation to large difference in thermal expansion coefficients between them epitaxial strain can be continuously tuned.^60^ It is determined by the difference between growth and room temperature, as well as the by the interface layer. Due to comparable thermal expansion coefficients of SrO and SrTiO_3_, similar increase in the in-plane unit cell size of SrO on Si is expected and corresponds to our experimental results.^61^

## 4. Conclusions

In the present study, SrO-assisted deoxidation and passivation of silicon surface was investigated with the focus on the interpretation of the 3D structure appearing as characteristic spotty RHEED pattern which precedes, but also coexists with 2 × 1 Sr-reconstruction of silicon surface. The results show that deoxidation of Si surface proceeds in uneven manner, which results in rough interface, while an increased surface mobility at high temperatures leads to formation of islands. The chemical composition analysis of sample with thicker SrO layer indicated formation of oxygen free Sr layer at the silicon surface, followed by a layer of silicate that is covered with 1–3 nm of SrO, whose crystallinity and (111) out of plane orientation has been confirmed by XRD. The *in situ* analysis of RHEED patterns indicate that 3D structure corresponds to SrO islands epitaxially grown on Si (001), with (100) and (111) out-of-plane orientations being simultaneously present. We presume that XRD detected only SrO (111) orientation as a more dominant component. The crystallographic relationship of SrO (100) to silicon substrate can be represented as: SrO (001)‖Si (001) out-of-plane and SrO (110)‖Si (100) in-plane, while mutual orientation of SrO (001) and SrO (111) can be represented as (0−22)[100]‖(0−22)[111]. With the knowledge of the composition, phases and exact crystallographic orientations of the topmost SrO layer, subsequent epitaxial growth of functional oxide layers can be studied and explored.

## Conflicts of interest

There are no conflicts to declare.

## Supplementary Material

RA-010-D0RA06548J-s001
