# Supplementary material for: Simultaneous heteroepitaxial growth of SrO (001) and SrO (111) during strontium-assisted deoxidation of the Si (001) surface
Source: RSC Adv. 2020 Aug 24;10(52):31261–70. doi: 10.1039/d0ra06548j (PMC9056433; doi:10.1039/d0ra06548j)
Supplement: RA-010-D0RA06548J-s001 [file RA-010-D0RA06548J-s001.pdf]

Electronic Supplementary Material (ESI) for RSC Advances.
This journal is © The Royal Society of Chemistry 2020

Simultaneous heteroepitaxial growth of SrO (001) and SrO (111) during strontium-assisted deoxidation of Si (001) surface

Zoran Jovanović[a,b,*], Nicolas Gauquelin[c], Gertjan Koster[a,d] Juan Rubio-Zuazo[e,f], Philippe Ghosez,[g] Jo Verbeeck[c], Danilo Suvorov[a] and Matjaž Spreitzer[a]

[a] Advanced Materials Department, Jožef Stefan Institute, Jamova 39, 1000 Ljubljana, Slovenia.

[b] Laboratory of Physics, Vinča Institute of Nuclear Sciences - National Institute of the Republic of Serbia, University of Belgrade, Belgrade, Serbia

[c] Electron microscopy for materials science (EMAT), University of Antwerp, Groenenborgerlaan 171, 2020 Antwerp, Belgium

[d] Faculty of Science and Technology and MESA+ Institute for Nanotechnology, University of Twente, P.O. Box 217, 7500 AE Enschede, the Netherlands

[e] SpLine, Spanish CRG BM25 Beamline at the ESRF (The European Synchrotron), F-38000, Grenoble, France.

[f] Instituto de Ciencia de Materiales de Madrid, Consejo Superior de Investigaciones Científicas (ICMM-CSIC), 28049, Madrid, Spain

[g] Theoretical Materials Physics, Q-Mat, CESAM, Université de Liège, B-4000 Liège, Belgium

- SUPPLEMENTARY INFORMATION -

[*] Corresponding author. E-mail addresses: zoran.jovanovic@ijs.si and zjovanovic@vin.bg.ac.rs

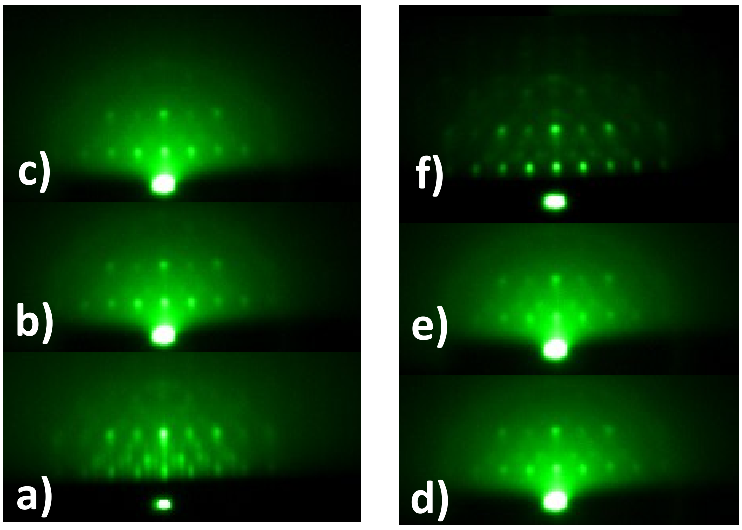

Figure S1. RHEED images of 3D structure. a) after initial deposition of 1 nm of SrO (in vacuum); b-e) deposition of additional 6 nm of SrO in argon via Route III; b) 2 nm, c) 4 nm, d) 5 nm, e) 6 nm. RHEED images are recorded in vacuum. f) The total thickness of 7 nm of SrO, recorded in vacuum. Incident azimuth in all cases was Si <100>.

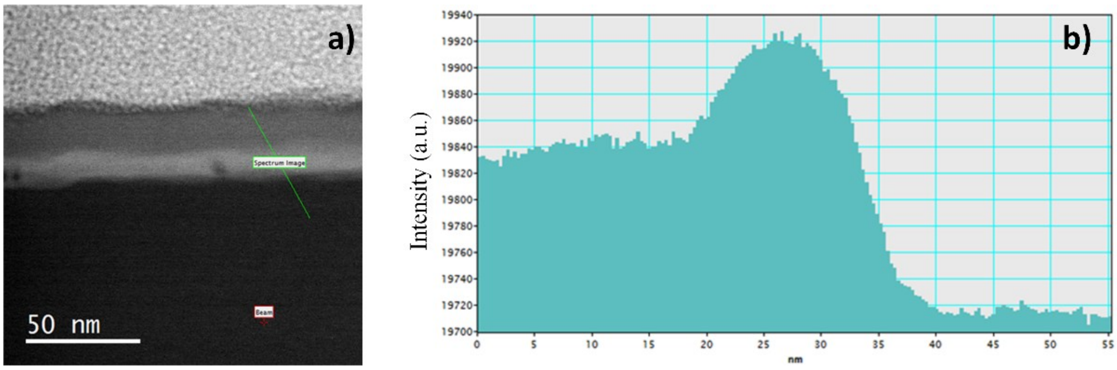

Figure S2. Area of the line scan **a)** and the corresponding HAADF profile **b)** of the $Si/SrSi_x/SrSiO_x/SrO_x/TiO_x$ sample.

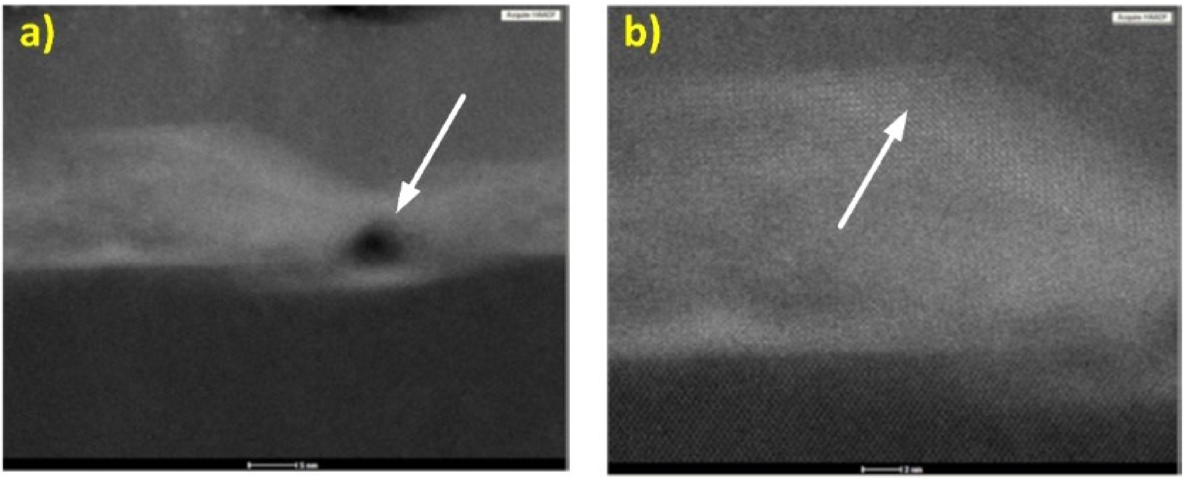

Figure S3. Electron-beam induced changes in Si/$SrSi_x$/$SrSiO_x$/SrO/TiO_x sample: a) Damaged region of the $SrSiO_x$ layer and b) formation of crystalline $SrTiO_3$ on the interface between SrO_x and TiO_x. Arrows point to areas of interest.

Calculation of reciprocal space distances

The determination of the distances in the reciprocal space is based on the feature of the RHEED method to probe the bulk of the crystal in the case of higher tilt angles. In this way, not only the distances in reciprocal space can be calculated but also the high-symmetry azimuthal direction can be identified. Because of the periodicity, the marked distances in Figure S4a and Figure S4d can be divided in two and four segments, respectively.

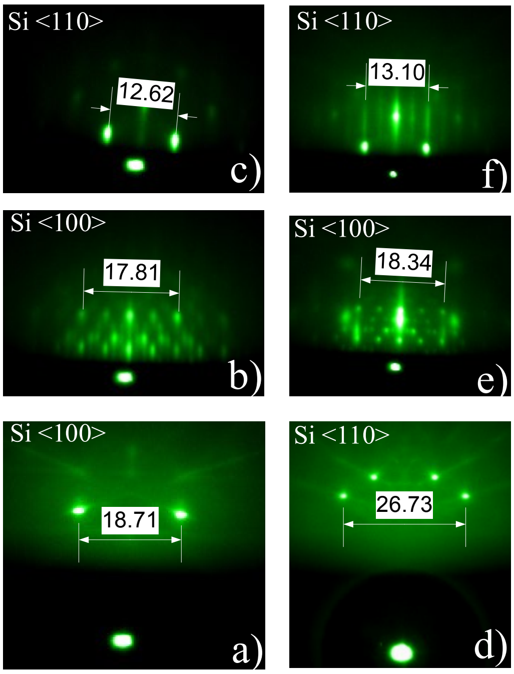

Figure S4: The RHEED patterns of Si (001)/SiO_2 substrate (**a** and **d**), 3D pattern (**b** and **c**) and mixture of a 3D pattern and 2× (1×) Sr-reconstruction of silicon surface (**e** and **f**). The distances were measured from the center of the spots, defined by the highest intensity. The azimuthal direction is marked in the upper left corner of RHEED images.

The length of segment is 9.3550 and 6.6825 for Figure S4a and Figure S4d, respectively, with ratio close to $\sqrt{2}$. Thus, it can be concluded that azimuthal directions in case of Figure S4a and Figure S4d are <100> and <110>, respectively. Since some RHEED patterns in our study contained both streaks (contribution from the smooth, 2×1 Sr-reconstructed silicon surface) and spots (contribution from 3D structure), to elaborate the reciprocal space distances the two approaches based on: i) surface lattice and ii) formalism of transmission electron microscopy can be used. For this purpose, we will use measured distances (Figure S4).

Surface lattice formalism

To calculate reciprocal space distances we first need to calculate screen constants from the measured distances of Si surface lattice (Figure S4a,d). For Si(001) surface lattice, whose lattice spacing is a=5.43 Å/√2, the reciprocal lattice spacing is (2π/a)=1.63642 Å^{-1}. Thus, the screen constants, k1 and k2, from Figure S4a and Figure S4d are, respectively:

$$\frac{18.71}{2} \cdot k_1 = 2.3142\ \text{Å}^{-1}, k_1 = 0.247381\ \text{Å}^{-1} \tag{1}$$

$$\frac{26.73}{4} \cdot k_2 = 1.6364\ \text{Å}^{-1}, k_2 = 0.244881\ \text{Å}^{-1} \tag{2}$$

These constants can be used to calculate reciprocal spacing of 3D structure and 2×1 Sr-reconstruction.

For 3D structure (Figure S4b,c):

$$\frac{17.81}{4} \cdot k_1 = 1.1015\ \text{Å}^{-1} \tag{3}$$

$$\frac{12.62}{2} \cdot k_1 = 1.5609\ \text{Å}^{-1} \tag{4}$$

For 2×1 Sr-reconstruction (Figure S4e,f):

$$\frac{18.34}{2} \cdot k_2 = 2.2456\ \text{Å}^{-1} \tag{5}$$

$$\frac{13.10}{4} \cdot k_2 = 0.8020\ \text{Å}^{-1} \tag{6}$$

In Figure S4e,f a spotty pattern of 3D structure can also be observed:

$$\frac{18.34}{4} \cdot k_2 = 1.1228\ \text{Å}^{-1} \tag{7}$$

$$\frac{13.10}{2} \cdot k_2 = 1.6040\ \text{Å}^{-1} \tag{8}$$

As can be observed, in the case of 2×1 Sr-reconstructed silicon surface, the calculated values (eqs. 5 and 6) are nicely matching the theoretical ones (2.3142 Å^{-1} and 0.8182 Å^{-1}) *i.e.* there is ~3% difference. As it was discussed in the main text, the most probable candidate that contributes to 3D structure is SrO. However, we can see that calculated reciprocal spacing of 3D structure (eqs. 3 and 4, eqs. 7 and 8) is larger than theoretical ones (0.388 Å^{-1} and 0.548 Å^{-1}). However, if we consider 3rd-order diffraction, then the values in eqs. 3 and 4, eqs. 7 and 8 should be divided by 3, hence giving the average values (0.371 Å^{-1} and 0.528 Å^{-1}, respectively) that are close to theoretical ones (0.388 Å^{-1} and 0.548 Å^{-1}).

Formalism of transmission electron microscopy

If we consider that spotty RHEED pattern is a result of transmitted e-beam, then diffraction pattern should follow selection rules based on structure factors. Let us apply the same approach for silicon substrate before (with native oxide) and after deoxidation (2×1 Sr-reconstructed surface).

Let us first calculate screen constants using the stated approach. Thus, the screen constants, k1 and k2, from Figure S4a and Figure S4d are, respectively:

$$\frac{18.71}{2} \cdot k_1 = 0.737\ \text{Å}^{-1}, k_1 = 0.078781\ \text{Å}^{-1} \tag{9}$$

$$\frac{26.73}{4} \cdot k_2 = 0.521\ \text{Å}^{-1}, k_2 = 0.077964\ \text{Å}^{-1} \tag{10}$$

These constants can be used to calculate reciprocal spacing of 3D structure and 2×1 Sr-reconstruction.

For 3D structure (Figure S4b,c):

$$\frac{17.81}{4} \cdot k_1 = 0.3508\ \text{Å}^{-1} \tag{11}$$

$$\frac{12.62}{2} \cdot k_1 = 0.4971\ \text{Å}^{-1} \tag{12}$$

For 2×1 Sr-reconstruction (Figure S4e,f):

$$\frac{18.34}{2} \cdot k_2 = 0.7149\ \text{Å}^{-1} \tag{13}$$

$$\frac{13.10}{4} \cdot k_2 = 0.2553\ \text{Å}^{-1} \tag{14}$$

In Figure S4e,f a spotty pattern of 3D structure can also be observed:

$$\frac{18.34}{4} \cdot k_2 = 0.3575\ \text{Å}^{-1} \tag{15}$$

$$\frac{13.10}{2} \cdot k_2 = 0.5107\ \text{Å}^{-1} \tag{16}$$

As can be observed, in the case of 2×1 Sr-reconstructed silicon surface, the experimental values (eqs. 13 and 14) are nicely matching the theoretical ones (0.738 Å^{-1} and 0.261 Å^{-1}). The same can be concluded if calculated values (eqs. 11, 12 and 15,16) are compared to theoretical one (Figure 5b). This method is more convenient since both surface and island contributions are considered based on structure factors characteristic for Si and SrO, because of which this method was used in the manuscript.

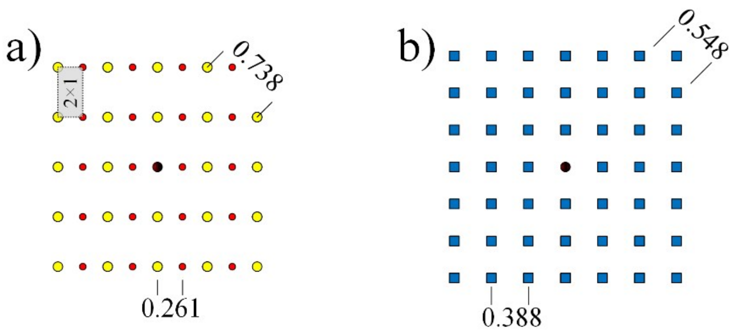

Figure S5. Reciprocal surface lattices of a) 2×1 reconstructed Si (001) surface and b) SrO (001) surface considering the structure factor. The distance between lattice points is scaled accordingly. The units are in Å^{-1}.
